# Licochalcone A-induced human gastric cancer BGC-823 cells apoptosis by regulating ROS-mediated MAPKs and PI3K/AKT signaling pathways

**DOI:** 10.1038/srep10336

**Published:** 2015-05-18

**Authors:** Wenjin Hao, Xuan Yuan, Lina Yu, Caixia Gao, Xiling Sun, Dong Wang, Qiusheng Zheng

**Affiliations:** 1 Binzhou medical University, Yantai, 264003, Shandong, China; 2 The Affiliated Hospital of Qingdao University, Qingdao, 266003, Shandong, China; 3 Qianfoshan Hospital of Shandong University, Jinan, 250014, China; 4 Key Laboratory of Xinjiang Endemic Phytomedicine Resources, Ministry of Education, School of Pharmacy, Shihezi University, Shihezi, 832002, Xinjiang, China

## Abstract

Both phosphatidylinositol 3-kinase (PI3K)/AKT and mitogen activated protein kinase (MAPK) signaling cascades play an important role in cell proliferation, survival, angiogenesis, and metastasis of tumor cells. In the present report, we investigated the effects of licochalcone A (LA), a flavonoid extracted from licorice root, on the PI3K/AKT/mTOR and MAPK activation pathways in human gastric cancer BGC-823 cells. LA increased reactive oxygen species (ROS) levels, which is associated with the induction of apoptosis as characterized by positive Annexin V binding and activation of caspase-3, and cleavage of poly-ADP-ribose polymerase (PARP). Inhibition of ROS generation by N-acetylcysteine (NAC) significantly prevented LA-induced apoptosis. Interestingly, we also observed that LA caused the activation of ERK, JNK, and p38 MAPK in BGC-823 cells. The antitumour activity of LA-treated BGC-823 cells was significantly distinct in KM mice *in vivo*. All the findings from our study suggest that LA can interfere with MAPK signaling cascades, initiate ROS generation, induce oxidative stress and consequently cause BGC cell apoptosis.

Gastric cancer is one of the most common malignant cancers with poor prognoses and cancer-related mortality rates worldwide^1^. Despite recent advances in targeted therapy and improved understanding of the biology and development of the malignancy, progress in the treatment of gastric cancer has been limited. Most newly diagnosed patients are incurable and have a lower survival rate^2^. Chemotherapy is an important therapeutic approach in the treatment of advanced gastric cancer, but its clinical applications are limited because of severe side effects. Although several new agents provide a better prognosis for patients with advanced gastric cancer, the chemosensitivity is low^3^.

Recently, increasing attention has been focused on the application of natural products in cancer chemopreventive therapy. Licochalcone A (LA, Fig. 1) is a flavonoid extracted from licorice root and has antiparasitic and anti-tumor activities^4^. Experimental evidences revealed that LA exerts chemoprevention by inducing cell cycle arrest and cell death. For example, LA caused G2 and late-G1 arrests in androgen-independent PC-3 prostate cancer cells^5^. LA has been found to induce apoptotic cell death in oral^6^, prostate^7^, ovarian^8^, and gastric cancer cells^1^. LA has also been reported to induce autophagy-related cell death by suppression of Bcl-2 expression and the mTOR pathway^9^. How LA mediates its anticancer effect is not completely understood. However, growing reports have shown that reactive oxygen species (ROS) generation plays a critical role in determining the LA-induced cancer cells apoptosis^10^,^11^,^12^.

Mitogen-activated protein kinases (MAPKs) are serine-threonine protein kinases that play an important role in the regulation of many cellular processes including cell growth and proliferation, differentiation, and apoptosis. MAPKs consist of growth factor-regulated extracellular signal-related kinases (ERKs), and the stress-activated MAPKs, c-jun NH2-terminal kinases (JNKs) and p38 MAPKs^13^. Prior studies have indicated that ROS can induce or mediate the activation of the MAPK pathways^14^,^15^ and the activation of ERK, JNK, and p38 MAPK signaling proteins were involved in apoptosis via ROS generation^16^,^17^,^18^,^19^,^20^. Besides MAPK pathways, the phosphatidylinositol 3-kinase (PI3K)/AKT signal transduction pathway, plays a pivotal role in cell survival and the enhanced protection of cancer cells from apoptosis during tumorigenesis^21^,^22^,^23^. However, few studies have shown that LA induces apoptotic effect through the modulation of the MAPKs and PI3K/AKT signaling pathways. In the present study, we found that LA inhibited of cell proliferation and caused BGC cell apoptosis via mediating ROS-mediated MAPKs and PI3K/AKT signaling pathways.

## Results

### LA inhibited cell proliferation in BGC cells

**Figure 2:**
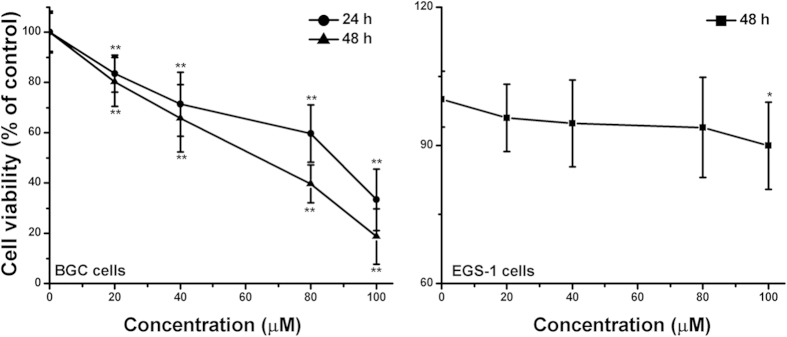
Effects of LA on cell viability in BGC cells. Cell viability was determined by using an MTT staining assay. Treatment of BGC cells with varying LA concentrations (0, 20, 40, 80 or 100 μM) for 24 or 48 h resulted in a significant concentration-dependent and time-dependent reduction in cell viability. The data represent the means ± S.D. of the three independent experiments. *P < 0.05, **P < 0.01 compared with the LA-untreated control group cell.

The effect of LA on cell proliferation was determined using MTT assay after 24 h or 48 h exposure, a significant concentration-dependent and time-dependent reduction in cell viability was observed, and the cell viability of 100 μM LA-treated BGC cells was decreased by 66.67% and 81.30% respectively (Fig. 2). While LA (20-80 μM) did not significantly affect the human gastric epithelial cells EGS-1. These results indicated that LA significantly suppressed the viability of human gastric cancer cells, with significantly lower toxicity against normal human gastric cells, suggesting that LA is a specific and effective inhibition agent against proliferation of human gastric cancer cells.

### LA induced oxidative stress in BGC cells

**Figure 3:**
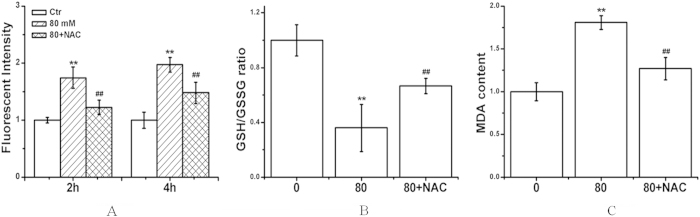
Effects of LA on ROS level, GSH/GSSG ratio and MDA production in BGC cells. The cells were treated with LA (0, 50, or 100 μM) with or without NAC (500 μM). (**A**) ROS level was determined via flow cytometry; (**B**) Oxidative stress was measured by GSH/GSSG ratio. (C) Oxidative damage to membrane lipid (lipid peroxidation) was measured via MDA levels. Control group (LA-untreated group) level was accepted to be “1.0”. Data are presented as the mean ± S.D. of the three independent experiments. *P < 0.05, **P < 0.01 compared with the control group; ^*#*^*P* < 0.05 compared with the LA group alone (80 μM).

In view of the significant growth inhibition of BGC cells induced by LA, we chose the concentrations of 100 μM for most of the subsequent assays. After the BGC cells were exposed to LA (100 μM) for 2 or 4 h, the intracellular ROS was significantly increased in a concentration-dependent manner compared with the control group (Fig. 3A). And GSH/GSSG ratio decreased obviously, accompanied with MDA (Malondialdehyde, a marker for oxidative stress, results from lipid peroxidation of polyunsaturated fatty acids. The degree of lipid peroxidation can be estimated by the amount of MDA) level increased (Fig. 3B,C). The antioxidant *N*-acetylcysteine (NAC, a precursor of glutathione, 500 μM) effectively prevented LA-induced ROS formation (2 or 4 h), GSH/GSSG ratio reduction and MDA level increase (Fig. 3).

### LA activated ERK, JNK, and p38 in BGC cells

**Figure 4:**
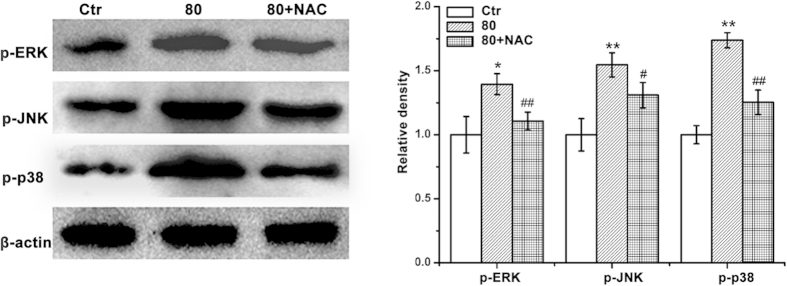
LA activated MAPKs signaling pathway in BGC cells. BGC cells were treated with LA (0 or 80 μM) with or without NAC (500 μM). (**A**) Then equal amounts of lysates were analyzed by Western blot analysis using antibodies against p-ERK, p-JNK and p-p38 (left), and quantitative analysis of p-ERK, p-JNK and p-p38 protein levels (right). The results were presented by cropped gels. Control group (LA-untreated group) level was accepted to be “1.0”. *P < 0.05,**P < 0.01 compared with the LCB-untreated control group; ^*#*^*P* < 0.05, ^*##*^*P* < 0.05 compared with the LA group alone (80 μM).

We next performed Western blot analysis to determine whether LA could induce the activation of MAPK cascades including JNK, ERK and p38 MAPK in BGC cells. As shown in Fig. 4, LA substantially induced the activation of ERK kinase, and slightly unregulated the phosphorylation of JNK and p38 MAPK in BGC cells. Next, we explored the potential role of ROS in LA-mediated ERK, JNK, and p38 MAPK activation in BGC cells. Results showed that the pretreatment of NAC drastically prevented the activation of these protein kinases induced by LA (Fig. 4).

### LA- mediated apoptosis in BGC Cells

**Figure 5:**
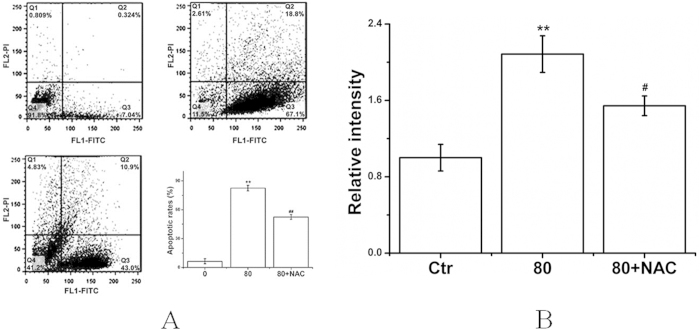
LA induced apoptosis in BGC cells. BGC cells were treated with or without the indicated amounts of LA for 48 h with or without NAC (500 μM). (**A**) Detection of apoptotic rates conducted via flow cytometry. (**B**) Caspase-3 activity was examined using fluorometric kit assay, with the relative intensity representing the result of caspase-3 activity. *P < 0.05, **P < 0.01 compared with the control group; ^*#*^*P* < 0.05, ^*##*^*P* < 0.01 compared with the LA group alone (80 μM).

Annexin V-FITC-PI double staining was used to detect phosphatidyl serine (PS) externalization, a hallmark of early apoptosis, to prove whether LA-induced apoptosis occurs. The apoptotic rates were markedly increased among LA-treated cells, as shown in Fig. 5A. The activation of caspase-3 provided additional support for the apoptosis induced by LA (Fig. 5B). Pretreatment with NAC (500 μM) effectively prevented LA-induced responses (Fig. 5).

### LA suppressed PI3K/AKT signaling cascade in BGC Cells

**Figure 6:**
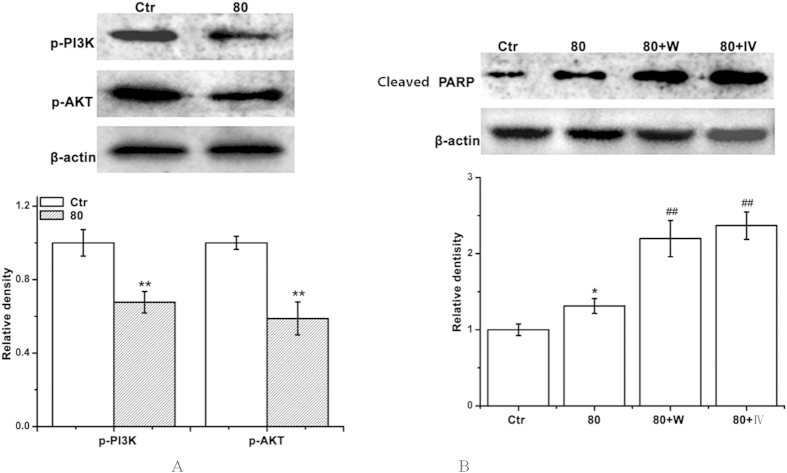
LA suppressed PI3K/AKT signaling pathway in BGC cells. BGC cells were treated with LA (0 or 80 μM) with or without PI3K and AKT inhibitors (1 mM wortmannin and 1 μM IV). (**A**) Then equal amounts of lysates were analyzed by Western blot analysis using antibodies against p-PI3K and p-AKT, and quantitative analysis of p-PI3K and p-AKT protein levels. The results were presented by cropped gels. (**B**) The effects of LA combination with PI3K and AKT inhibitors on activation of PARP protein. Control group (LA-untreated group) level was accepted to be “1.0”. *P < 0.05,**P < 0.01 compared with the LCB-untreated control group; ^*#*^*P* < 0.05, ^*##*^*P* < 0.01 compared with the LA group alone (80 μM).

The PI3K/AKT-mediated signal pathway is significant in regulating apoptosis. We first investigated whether LA down-regulated PI3K/AKT activation in BGC cells. As shown in Fig. 6A, activation of both PI3K and AKT was suppressed in LA-treated BGC cells. We further investigated the effects of LA on apoptosis by combination with PI3K and AKT inhibitors (1 mM wortmannin and 1 μM IV). Compared with LA alone, the combination of LA with these inhibitors activated PARP protein (Fig. 6B). The data indicated that the inhibition of PI3K/AKT signaling cascade by LA leaded to the suppression of cell proliferation in human gastric cancer BGC cells.

### Inhibition effects of LA on tumor growth *in vivo*

**Figure 7:**
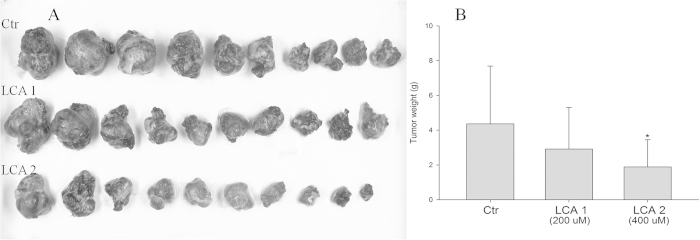
Inhibition of LA on tumour growth *in vivo*. KM mice bearing gastric cancer BGC-823 cells were administrated with LA by intratumoral injection every two day. The implanted sarcomas were separated and weighed on day 11. (**A**) Images of the tumour morphology. (**B**) The quantitative results of tumor growth-inhibition. Data are presented as mean ± S.D. from 10 individual treatments. * p < 0.05 compared with LA-untreated control group cell.

Based on the encouraging findings that LA inhibited gastric cancer BGC-823 cells growth *in vitro*, we used BGC-823 tumor models to investigate whether LA could suppress tumor progression *in vivo*. The BGC-823 gastric carcinoma in KM mice was used as an *in vivo* model to evaluate the effects of LA on tumor growth. As shown in Fig. 7, tumor growth inhibition was significantly distinct in mice treated with LA at 400 μM, compared with mice treated with normal saline (Fig. 7A), while the inhibition rate was 49.88% and 57.84% when treated with LA (200 μM) and LA (400 μM), respectively.

## Discussion

Even though LA has been known to suppress the proliferation of a wide variety of cancer cells and exert cancer chemopreventive activity by inducing the apoptosis of cancer cells^4^,^24^. The precise mechanisms underlying the apoptotic cell death caused by LA are mostly unclear. The most important findings of this study were that LA reduced the cell viability, enhanced ROS, targeted MAPKs signaling pathway and induce apoptosis, and the inactivation of constitutive PI3K/AKT also implicated in LA-treated human gastric cancer BGC-823 cells apoptosis.

Reactive oxygen species (ROS), represented by superoxide, hydrogen peroxide and hydroxyl radicals, ROS are oxygen-derived free radicals, have been known to lead to DNA lesions, protein oxidation and lipid peroxidation, also implicated in the initiation and promotion of multistep carcinogenesis^25^,^26^. However, chemotherapeutic agents are used to induce ROS-mediated apoptosis in different tumor cells^27^. A number of anti-cancer drugs exert their effect by causing DNA damage which leads to apoptosis induction. Direct involvement of ROS overproduction was also demonstrated in LA-induced BGC cell apoptosis, evidenced by intracellular ROS production, GSH/GSSG ratio decrease, and MDA level increase (Fig. 3). ROS generation can also concurrently activate MAPKs pathway in multiple cell types, we therefore examined the possibility of whether MAPKs pathway was involved in LA-induced BGC cells apoptosis. The results showed that LA could inactivate phospho-AKT, activated of phospho-p38, phospho-ERK in BGC cell. The generation of ROS in response to LA was further supported by the finding that pretreatment with NAC blocked the ROS-mediated MAPK activation (Fig. 4) and prevented LA-induced apoptosis (Fig. 5) in cancer cells. Thus, it is most likely that the induction of MAPK activation can contribute to the suppression of apoptosis in part upon LA treatment in BGC cells.

The PI3K/AKT is the major anti-apoptotic pathway that confers the survival advantage and resistance of cancer cells against various chemotherapeutic agents^28^. In numerous cancer tissues and cells, this pathway is overactive, reducing apoptosis and allowing proliferation. The present study identified that LA treatment significantly inhibited the PI3K and AKT activation, and combination of LA with wortmannin (PI3K inhibitor) enhanced LA-induced BGC cells apoptsis, which consistent with the combination of AKT inhibitor IV (Fig. 6). The results indicated that LA was able to trigger apoptosis of the gastric cancer cells through the PI3K/Akt-mediated pathway. Further, the BGC-823 tumor models were established to investigate whether LA could suppress tumor progression *in vivo*. Results revealed LA could inhibit the tumor growth *in vivo* (Fig. 7).

In conclusion, the results demonstrated that LA could induce ROS-mediated MAPKs activation, inhibit PI3K/AKT signaling pathway, and lead to BGC cell apoptosis. Thus, LA is a potential therapeutic agent for further development for management of human gastric cancer.

### Ethics statement

All experimental protocols were approved by local animal care and use committee. The methods were carried out in accordance with the approved guidelines.

## Materials and methods

The methods were carried out in accordance with the approved guidelines.

### Reagents

LA (purity ≥ 98%) was purchased from Tianjin Zhongxin Pharmaceutical Group Co., Ltd. (Tianjin, China). Culture medium (RPMI 1640), dimethylsulfoxide (DMSO), Hoechst 33258, N-acetylcysteine (NAC), Annexin V/PI apoptosis kit, and molecular Probes 2’,7’-dichlorodihydrofluorescein diacetate (H_2_DCFDA) were purchased from Sigma (St. Louis, Missouri, USA). Fetal bovine serum (FBS) was purchased from Tianjin Hao Yang Biological Manufacture Co., Ltd. (Tianjin, China). The antibodies used in this study were purchased from Santa Cruz Biotechnology Inc. (Santa Cruz, CA, USA). Penicillin and streptomycin were obtained from Shandong Sunrise Pharmaceutical Co., Ltd. (Shandong, China). LA was dissolved in DMSO and diluted with fresh medium to achieve the desired concentration. The final concentration of DMSO did not exceed 0.2% in the fresh medium, and DMSO at this concentration had no significant effect on the cell viability. Unless indicated otherwise, the other reagents were purchased from Sigma.

### Cell line and cell culture

Human gastric cancer cell line BGC and the human gastric epithelial cell line GES-1 were purchased from Cell Bank of the Committee on Type Culture Collection of the Chinese Academy of Sciences (Shanghai, China). The cells were maintained in RPMI 1640 medium supplemented with 10% FBS, 100 U/mL penicillin and 100 μg/mL streptomycin at 37 °C with 5% CO_2_. The cells were split every 3 days and were diluted every day before each experiment.

### Cell viability assay

Cell viability was measured by the MTT [3-(4,5-dimethylthiazol-2-yl)-2, 5-diphenyl-tetrazolium bromide] assay^29^. In brief, cells were washed with fresh media and cultured in 96-well plates (5 × 10^3^ cells/well) and then incubated with LA (0, 20, 40, 80 or 100 μM) for 24 or 48 h. After incubation, the medium was aspirated and fresh medium containing 10 μL of 5 mg/mL MTT was added. After 4 h, the medium was removed and replaced with blue formazan crystal dissolved in 100 μL dimethyl sulfoxide (DMSO). Absorbance at 570 nm was measured using a fluorescent plate reader (Millipore Corp., Bedford, MA, USA). The data were expressed as percent cell viability compared with control group.

### Detection of intracellular reactive oxygen species (ROS) level

To determine the intracellular level of ROS we used fluorogenic probe 2’,7’-dichlorodihydrofluorescein diacetate (H_2_DCFDA)^30^. Briefly, the cells were incubated with the indicated concentrations of LA with or without NAC (500 μM) for 0.5, 1, 2 or 4 h. Cells were then washed in phosphate buffered saline (PBS) and incubated with 30 μM H_2_DCFDA at 37 °C for 30 min. Stained cells were washed, resuspended in PBS, and analysed using a FACStar flow cytometer (Becton Dickinson, New Jersey, USA). The fluorescence of the stained cells were analysed by flow cytometry. Each group was acquired more than 10 000 individual cells.

### GSH/GSSG ratio measurement

Oxidative stress was assessed through GSH/GSSG ratio measurement. The concentrations of total glutathione (T-GSH), reduced glutathione (GSH), and oxidized disulfide (GSSG) were measured via an enzymatic method. T-GSH was assayed using 5,5-dithio-bis(2-nitrobenzoic) acid (DTNB)-GSSG reductase recycling. GSSG was measured by measuring 5-thio-2-nitrobenzoic acid (TNB) produced from the reduced GSH reaction with DTNB. The TNB formation rate was measured at 412 nm. The reduced GSH concentration was obtained by subtracting GSSG from T-GSH^31^,^32^.

### Malondialdehyde (MDA) content measurement

MDA formation, a substance produced during lipid per-oxidation, was determined using the thiobarbituric acid reactive substance (TBARS) test. BGC cells were har-vested after ISL exposure with or without NAC for 48 h, and aliquots of 10% supernatant were incubated with 0.8% TBA. The mixture was heated in 95 °C water bath for 1 h. Afterward, n-butanol and pyridine (15:1, V/V) were added, after which the mixture was centrifuged. The organic phase was collected to measure the fluorescence at excitation and emission wavelengths of 515 and 553 nm, respectively. A standard curve was generated using 1,1,3,3-tetramethoxypropane. MDA content was expressed as nmol/mg protein. The protein content was measured using the method of Bradford^33^.

### Detection of cell apoptotic rates by flow cytometry

LA induced apoptosis in BGC cells was determined by flow cytometry using the Annexin V-FITC Apoptosis Detection Kit following the manufacturer’s instructions. Briefly, 1.5 × 10^5^ cells/mL were incubated with LA with or without NAC (500 μM) for 48 h. Afterwards, the cells were washed twice with ice-cold PBS, and then 5 μL of annexin V-FITC and 1 μL of PI (1 mg/mL) were then applied to stain cells. The stained cells were analyzed using a flow cytometer^34^.

### Measurements of caspase-3 activity

Caspase-3 activity was assessed using a fluorometric kit assay according to the manufacturer’s instructions (BioVision). Briefly, Cells were lysed and proteins (20 μg) were incubated with caspase-3 substrate DEVD-AFC (50 μg) at 37 °C for 1 h to 2 h. Samples were transferred in black bottom 96-well microplates and read in the fluorescent plate reader (Milli-pore). The non-cleaved (blue) and cleaved (green) substrate emissions were 400 and 505 nm, respectively. Control reactions were performed without proteins in wells and by omitting the substrate^35^.

### Western blot analysis

The soluble lysates (15 μL per lane) were subjected to 10% sodium dodecyl sulfate-polyacrylamide gel electrophoresis (SDS-PAGE), and then transferred onto the nitrocellulose membranes (Amersham Biosciences, New Jersey, USA) and blocked with 5% nonfat milk in Tris-buffered saline with Tween (TBST) for 2 h at room temperature. Membranes were incubated with primary antibody (anti-phospho-AKT (p-AKT) antibody (1:200) sc-135650, anti-phospho-p38 (p-p38) antibody (1:200) sc-7975-R, anti-phospho-ERK (p-ERK) antibody (1:200) sc-7976, anti-phospho-JNK (p-JNK) antibody (1:200) sc-6254, anti-phospho-PI3K (p-PI3K) antibody (1:200) sc-12929, anti-poly (ADP-ribose) polymerase (PARP) antibody (1:200) sc-56196, anti-β-actin antibody (1:2000) sc-47778) (Santa Cruz Biotechnology, Santa Cruz, CA, USA) at 4 °C overnight and then incubated with the appropriate horseradish peroxidase-conjugated secondary antibody. Western blots were developed by using enhanced chemiluminescence (ECL, Thermo Scientiic) and were exposed on Kodak radiographic film. All of the Western blot gels were performed under the same experimental condition.

### Animal preparation

SPF KM mice, aged 6–8 weeks and weighted from 13 to 15 g, were obtained from the Medical Laboratory Animal Center (SDXK (Xin) 2011-004), Xinjiang Medical University, Xinjiang, China. The mice were maintained under standard animal care conditions (22 ± 3 °C and 60% humidity) for 14 days, with a commercial standard mouse cube diet (Shihezi University Laboratory Animal Center, Xinjiang, China) and water *ad libitum*. All animal procedures were performed in accordance with the relevant guidelines, and were approved by the Laboratory Animal Center in Shihezi University.

### *In vivo* antitumour activity assay

The murine transitional cell carcinoma cell line BGC-823 (2 × 10^6^ /mL, 100 μL) was injected subcutaneously into the flank of KM mice. Tumor formation in KM mice was monitored. Subcutaneous tumors induced by BGC-823 cells in KM mice were randomly divided into two treatment groups (10 of each group). One week after inoculation, the mice were given of 200 and 400 μM of LA respectively by intratumoral injection every two day. Control mice were given the same volume of normal saline. The animals were sacrificed at 30 days after cancer cells inoculation. The implanted sarcomas were separated and weighed, then the tumor inhibition rate (TIR) was calculated according to the following formulate: TIR (%) = (WC − WE)/WE × 100%. WC: Mean tumor weight in control group; WE: Mean tumor weight in tested groups respectively; >30% was regarded as having inhibitory effect.

### Statistical analysis

The data were presented as means ± S.D. from at least three independent experiments and evaluated through the analysis of variance (ANOVA) followed by student’s *t*-test. The values of *P* *<* 0.05 were considered statistically significant. The analyses were performed by using the Origin 8.0 software (Origin Lab Corporation, Northampton, MA, USA).

## Author Contributions

W.H., X.Y., L.Y., C.G., X.S., D.W. and Q.Zh. designed the study. W.H., X.Y., L.Y. and C.G. performed experiments, prepared figures. W.H., X.Y., L.Y., C.G., X.S. and Q.Zh. analysed the data. W.H., X.Y., L.Y., C.G., X.S., D.W. and Q.Zh. wrote and discussed all sections of the manuscript. All authors reviewed the manuscript.

## Additional Information

**How to cite this article**: Hao, W. *et al.* Licochalcone A-induced human gastric cancer BGC-823 cells apoptosis by regulating ROS-mediated MAPKs and PI3K/AKT signaling pathways. *Sci. Rep.* **5**, 10336; doi: 10.1038/srep10336 (2015).

## Figures and Tables

**Figure 1:**
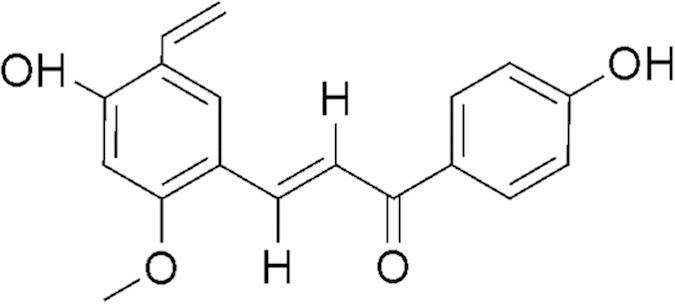
Chemical structure of licochalcone **A**.
